# Public and outpatients’ awareness of calling emergency medical services immediately by acute stroke in an upper middle-income country: a cross-sectional questionnaire study in greater Gaborone, Botswana

**DOI:** 10.1186/s12883-022-02859-z

**Published:** 2022-09-14

**Authors:** Ookeditse Ookeditse, Kebadiretse K. Ookeditse, Thusego R. Motswakadikgwa, Gosiame Masilo, Yaone Bogatsu, Baleufi C. Lekobe, Mosepele Mosepele, Henrik Schirmer, Stein H. Johnsen

**Affiliations:** 1 Department of Physical Medicine and Rehabilitation, Division of Neurorehabilitation Medicine, Trust hospital in Vestfold, Kysthospitalet Sykehuset, P.O. Box 2168, 3103 Tønsberg, Norway; 2 Department of Internal Medicine, Sidilega Private Hospital, Gaborone, Botswana; 3 Faculty of Medicine, University of Botswana, Gaborone, Botswana; 4 Department of Family Medicine and Occupational Medicine, Sidilega Private Hospital, Gaborone, Botswana; 5 Department of Family Medicine, Larvik County Acute and Emergency Clinic, Larvik, Norway; 6 Division of Family Medicine, Nanset Medical Clinic, Larvik, Norway; 7 Princess Marina Referral Hospital, Gaborone, Botswana; 8 UIT The Arctic University of Norway, Institute of Clinical Medicine, Tromsø, Norway; 9 Department of Cardiology, Akershus University Hospital, Lørenskog, Norway; 10 University of Oslo, Institute of Clinical Medicine, Oslo, Norway; 11 Department of Neurology, University Hospital of North Norway, Tromsø, Norway

**Keywords:** Stroke symptoms, Stroke risk factors, Outpatients, Public, Awareness, Emergency medical services, Thrombolytic therapy

## Abstract

**Objectives:**

In this cross-sectional study from Botswana, we investigated awareness of calling emergency medical services (EMS) and seeking immediate medical assistance by acute stroke among stroke risk outpatients and public.

**Method:**

Closed-ended questionnaires on awareness of calling EMS and seeking immediate medical assistance by acute stroke, were administered by research assistants to a representative selection of outpatients and public.

**Results:**

The response rate was 96.0% (93.0% for public (2013) and 96.6% for outpatients (795)). Public respondents had mean age of 36.1 ± 14.5 years (age range 18–90 years) and 54.5% were females, while outpatients had mean age of 37.4 ± 12.7 years (age range 18–80 years) and 58.1% were females.

Awareness of calling EMS (78.3%), and of seeking immediate medical assistance (93.1%) by stroke attack was adequate. For calling EMS by acute stroke, outpatients had higher awareness than the public (*p* < 0.05) among those with unhealthy diet (90.9% vs 71.1%), family history of both stroke and heart diseases (90.7% vs 61.2%), no history of psychiatric diseases (93.2% vs 76.0%) and sedentary lifestyle (87.5% vs 74.8%).

Predictors of low awareness of both calling EMS and seeking immediate medical assistance were no medical insurance, residing/working together, history of psychiatric diseases, and normal weight.

Male gender, ≥50 years age, primary education, family history of both stroke and heart diseases, current smoking, no history of HIV/AIDS, and light physical activity were predictors of low awareness of need for calling EMS.

**Conclusion:**

Results call for educational campaigns on awareness of calling EMS and seeking immediate medical assistance among those with high risk factor levels.

**Supplementary Information:**

The online version contains supplementary material available at 10.1186/s12883-022-02859-z.

## Highlights

• This is the first study comparing awareness of calling EMS among outpatients and public in sub-Saharan Africa

• Awareness of calling EMS or seeking immediate medical services by acute stroke was adequate among both outpatients and public

• Predictors of low awareness of both calling EMS and seeking immediate medical assistance were no medical insurance, residing/working together, history of psychiatric diseases, and normal weight

• Results call for educational campaigns on awareness of calling EMS/ seeking immediate medical assistance by stroke.

**Supplementary Information:**

The online version contains supplementary material available at 10.1186/s12883-022-02859-z.

## Introduction

Stroke was the second largest cause of death and third largest cause of disability-adjusted life-years (DALYs) lost globally in 2019 according to World Health Organization (WHO) estimates [1]. The burden of stroke shifted from high-income countries (HIC) to low- and middle-income countries (LMIC) already in 2010 [2]. The incidence of stroke decreased in most regions from 1990 to 2016 while it increased in east Asia and southern sub-Saharan Africa (SSA) [3]. Globally, the highest age-standardized incidence of stroke is in Africa [4].

Thrombolysis has shown to be an effective treatment for acute ischemic stroke within 4.5 h of onset due to revascularization, improving clinical outcome and dependency in DALYs [5–7]. The emergence of intravenous thrombolysis and thrombectomy has increased the focus on stroke as an urgent and emergency disease due to time-dependent therapies, and that benefit increases when onset-to-treatment-time (OTT) decreases [8, 9]. Several studies revealed patient’s delay as barrier to thrombolytic therapy as most acute stroke patients arrive late to hospital as none or only a few use emergency medical transport (EMT), contact family members or the family doctor [10–26]. According to several studies conducted in Europe and the United States of America, 50–70% of patients are transported to hospital by EMT [24, 27–33].

Decreasing time from stroke onset to hospital arrival might increase the proportion of patients available for therapy [34], hence improving outcomes. Use of EMS shorten the time to diagnosis and treatment and increase the frequency of revascularisation [27–33, 35–40]. Most of the previous studies have assessed likelihood of calling EMS when experiencing stroke (symptoms) but have never assessed awareness of the urgency of calling EMS immediately.

## Objectives

1. To assess awareness of calling EMS, and awareness of seeking immediate medical assistance by acute stroke among public and outpatients in Botswana.
2. To assess if respondents’ sociodemographic and stroke risk factors influence awareness of calling EMS.

## Methods

### Study design and population

In this cross-sectional questionnaire survey study, participants were recruited from Botswana, which is an upper middle-income country under LMIC in SSA. The study purposively sampled a variety of respondents from the public with/without stroke risk factors, and outpatients with stroke risk factors in greater Gaborone. Respondents from the public were recruited from their homes or workplaces. Outpatients from both primary and secondary healthcare facilities while waiting for or after their medical consultation.

Trained research assistants interviewed respondents verbatim. Each interviewer conducted a standardized, structured, one-to-one interview, according to a multi-sectioned questionnaire designed to guide interview and avoid bias. For the public, no more than 2 respondents from same family/compound/ company were interviewed. The interviewer intervened only if asked to clarify any question, without giving correct answers. We sampled only odd numbers for outpatients in a queue at healthcare facilities and households for the public in each area. For the public, we further sampled from various socio-economic levels i.e., high, moderate and low socio-economic areas within greater Gaborone.

### Data collection instrument

The survey instruments were adapted from previous surveys [41–43] with some modifications to reflect the recent American Heart Association/American Stroke Association (AHA/ASA) guidelines and European Stroke Organization guidelines [44, 45]. We tested the questionnaire in a pilot study with a sample of 25 respondents and changes were made in the wording of questions based on the result of the pilot study accordingly. The questionnaire instruments were anonymous, electronic-based, written, and administered in English or local language (Setswana), closed-ended in nature and categorized into sections.

The questionnaire was divided into 4 sections (eFigure 1). Section 1 included respondents’ sociodemographic factors. Variables included were age (18–34 years, 35–49 years or ≥ 50 years), gender, education (none/ unknown/ primary, secondary or tertiary), medical insurance, and residing or working status (same family/working place).

Section 2 covered responses to acute stroke, and individual stroke symptoms as described below. We assessed awareness of respondents for seeking immediate emergency care and for calling EMS in response to a perceived stroke and specified individual stroke symptoms:

“What would you do when you suspect you are having stroke?” Responses included call 991, 911/997/8 or another emergency number, call a family member, the pastor, contact a traditional doctor, go to the pharmacy, no idea/ nothing or wait and see. Answers were dichotomized into calling EMS vs other options. EMSs are first responders in the country. They comprise of a physician, nurses and paramedics. They offer services that include stabilising and transporting acute sick patients to hospitals in the country. EMS services in the county are provided by MedRescue services, Emergency assist, and Boitekanelo emergency.

“If you get stroke, how long would you take before seeking medical assistance?” Answers included immediately, 7 h, 1 day, 3 days, 1 week, or no idea/no answer. We then dichotomized the responses to seeking medical assistance immediately vs other options.

We also assessed the level of medical care respondents would seek if they got specified stroke symptoms in a closed-ended question as follows: “Which of the following would you do first if you suspected that you are having one of the following e.g., acute weakness on one side of the body?” Answers included call 911 EMS, contact medical clinic, no idea, nothing or wait and see. We then categorized the responses into 3: calling EMS vs medical clinic vs other options.

Each correct answer in section 2 scored 1 point and was considered being aware. Each incorrect, unanswered or unknown answer scored 0 point and was considered being unaware.

Section 3 and 4 comprised respondents’ stroke risk factors and sources of stroke information respectively.

#### Respondents’ stroke risk factors

Included hypertension, family history of stroke, heart diseases or both (at least in one family member in the first generation), history of Human immunodeficiency virus (HIV/AIDS): (yes or no), psychiatric diseases (depression/ anxiety): (yes or no), smoking (non-smokers, former, or current), alcohol drinking (non-drinkers, former, or current), dietary status (perceived healthy or unhealthy) and one or more of four less common cardiovascular risk factors (CVDS: diabetes, dyslipidemia, prior stroke, or heart diseases). Lastly, perceived and calculated BMI categories (underweight, normal, overweight or obesity), and physical activity (none, light, moderate or high physical intensity).

Current smokers were individuals who smoked at least one tobacco product daily in the previous 12 months, including those who had quit within the past year. Former smokers had quit more than 1 year earlier, while non-smokers had never used tobacco products.

Current drinkers were individuals who drank alcohol regularly in the previous 12 months, including those who had quit within the past year. Former drinkers had quit more than 1 year earlier, while non-drinkers had never used alcohol.

Information on physical activity at work, at home, during recreational or sport, and leisure-time activities was obtained using part of the International Physical Activity Questionnaire with comparable variables [46]. Questions were asked about frequency of regular specific activities the individual performs that increase breathing rate for at least 10 minutes: the total duration per day, the number of days in a week, and whether they perceived the activity as heavy, moderate, light, or no activity. For everyone, the recorded activities were converted to metabolic equivalent task (MET)-minutes per week (min/wk) [46]. Individuals participating in activities of less than 3.5 MET-min/wk. were classified as no activity (sedentary lifestyle), 3.5- < 600 MET-min/wk. as low, 600- < 3000 MET-min/ wk. as moderate, and > 3000 MET-min/wk. as high level of physical activity.

Participants were asked if they perceived their weight as underweight, normal, overweight or obese. Weight and height were measured, BMI calculated, and classified as defined by the World Health Organization (WHO) and National Institutes of Health (NIH) i.e., underweight as BMI < 18·5 kg/m^2^, normal BMI 18.5- < 25 kg/m^2^, overweight 25- < 30 kg/m^2^, and obesity as ≥30 kg/m^2^ [47, 48]. Height was measured twice to the nearest millimeter using a fixed plastic, non-elastic stadiometer, and average height calculated. Body weight was measured in kilograms (to the second decimal place) by a self-zeroing digital weight scale for adults dressed in light clothes without shoes. Safeway self-zeroing digital weight scales (Safeway Scale, South Africa) were used after calibration.

#### Sources of stroke information

We assessed respondents’ source of stroke information in a closed-ended question with six answers as follows, “Where did you get information about stroke?” Answers included family/ friends, television/ radio, newspaper/ magazines, doctors/ nurses, social media (internet, Facebook, Instagram, WhatsApp), and others (own experience, school, or patients).

### Statistical analysis

Continuous variables were expressed as mean ± standard deviation (SD). Categorical and ordinal variables were expressed as absolute frequency (n) and proportion (%) of the overall sample or subgroups. Outpatients and public groups’ awareness of stroke were compared using chi-square test.

Mann-Whitney U/ Kruskal-Wallis H was used to determine predictors of calling EMS or seeking immediate medical assistance by acute stroke among respondents’ sociodemographic and stroke risk factors. Bonferroni correction was used for multiple comparisons. Statistical tests were two-tailed and reported statistically significant at *p* < 0.05. All statistical analyses were completed using SPSS 25 statistical software (SPSS Inc., Chicago, Illinois, USA).

## Results

We interviewed 2987 respondents in a cross-sectional study in greater Gaborone from June–October 2019, excluded 179 participants (151 from the public and 28 outpatients) because of missing either consent or substantial information (eFigure 2). We had a valid response of 2808 respondents (94.0%), comprising 2013 from the public (93.0%) and 795 outpatients (96.6%). The public’s mean age was 36.1 ± 14.5 years (age range 18–90 years) and 54.5% were females, while for outpatients the mean age was 37.4 ± 12.7 years (range 18–80 years) and 58.1% were females. For more information on respondents’ characteristics, see Table 1.

**Table 1:** Sociodemographic and stroke risk factors among respondents

	Total	Public	Outpatients	
	*n* = 2808	*n* = 2013	*n* = 795	***p***
	n (%)	n (%)	n (%)	
**Sociodemographic factors**				
**Gender**				
Female	1559(55.5)	1097(54.5)	462(58.1)	0.416
Male	1249(44.5)	916(45.5)	333(41.9)	0.356
**Age category (years)**	missing 6	missing 5	missing 1	
18–34	1501(53.6)	1118(55.7)	383(48.2)	0.082
35–49	842(30.0)	588(29.3)	254(32.0)	0.409
> 50	459(16.4)	302(15.0)	157(19.8)	0.055
**Education level**				
Primary, unknown, none	367(13.1)	252(12.5)	115(14.5)	0.371
Secondary	1518(54.1)	1113(55.3)	405(50.9)	0.314
Tertiary	923(32.9)	648(32.2)	275(34.6)	0.483
**Medical insurance**				
Yes	420(15.0)	0(0.0)	420(52.8)	< 0.001
No, unknown	2388(85.0)	2013(100.0)	375(42.7)	< 0.001
**Marital status**				
Married/cohabiting	982(35.0)	728(36.2)	254(31.9)	0.223
Others	1826(65.0)	1285(63.8)	541(68.1)	0.381
**Residing/working together**				
Yes	1121(39.9)	1011(50.2)	110(13.8)	< 0.001
No	1687(60.1)	1002(49.8)	685(86.2)	< 0.001
**Self-reported risk factors**				
**History of hypertension**				
Yes	276(9.8)	141(7.0)	135(17.0)	< 0.001
No, unknown	2532(90.2)	1872(93.0)	660(83.0)	0.073
**History of CVDS**				
Yes	196(7.0)	117(5.8)	79(9.9)	0.012
No	2612(93.0)	1896(94.2)	716(90.1)	0.468
**Family history of stroke/heart diseases**				
Stroke	372(13.2)	313(15.5)	59(7.4)	< 0.001
Heart diseases	347(12.4)	227(11.3)	120(15.1)	0.075
Both heart diseases and stroke	389(13.9)	227(11.3)	162(20.4)	< 0.001
None	1700(60.5)	1246(61.9)	454(57.1)	0.294
**BMI**				
Underweight	53(1.9)	33(1.6)	20(2.5)	0.302
Normal, unknown	2429(86.5)	1860(92.4)	569(71.6)	< 0.001
Overweight	215(7.7)	111(5.5)	104(13.1)	< 0.001
Obesity	111(4.0)	9(0.4)	102(12.8)	< 0.001
**Healthy dietary status**				
No, unknown	1119(39.9)	802(38.8)	317(39.9)	0.993
Yes	1689(60.1)	1211(60.2)	478(60.1)	0.994
**Alcohol consumption**				
Current	668(23.8)	406(20.2)	262(33.0)	< 0.001
Former	46(1.6)	24(1.2)	22(2.8)	0.054
No, unknown	2094(74.6)	1583(78.6)	511(64.3)	0.004
**Smoking status**				
Current	337(12.0)	182(9.0)	155(19.5)	< 0.001
Former	43(1.5)	22(1.1)	21(2.6)	0.051
No, unknown	2428(86.5)	1809(89.9)	619(77.9)	0.027
**Intensity of physical activity**				
None, unknown	2157(76.8)	1582(78.6)	575(72.3)	0.224
Light	105(3.7)	62(3.1)	43(5.4)	0.054
Moderate	483(17.2)	337(16.7)	146(18.4)	0.513
High	63(2.2)	32(1.6)	31(3.9)	0.016
**History of HIV/AIDS**				
Yes	569(20.3)	289(14.4)	280(35.2)	0.001
No, unknown	2229(79.4)	1724(85.6)	515(64.8)	< 0.001
**History of psychiatric diseases**				
Yes	89(3.2)	0(0)	89(11.2)	< 0.001
No	2719(96.8)	2013(100.0)	706(88.8)	0.052
**Calculated risk factors**				
**Physical activity intensity (Met min/week)**				
Physical inactive, unknown	2169(77.2)	1585(78.7)	584(73.5)	0.307
Low (> 3,5–600)	112(4.0)	66(3.3)	46(5.8)	0.045
Moderate (> 600–3000)	436(15.5)	290(14.4)	146(18.4)	0.098
High (> 3000)	91(3.2)	72(3.6)	19(2.4)	0.244
**BMI**				
Underweight (< 18.5)	105(3.7)	85(4.2)	20(2.5)	0.113
Normal, unknown (18.5 < 25)	1178(42.0)	904(44.9)	274(34.5)	0.005
Overweight (25 < 30)	669(23.8)	458(22.8)	211(26.5)	0.197
Obesity (> 30)	856(30.5)	566(28.1)	290(36.5)	0.013

*NA* not applicable, *CVDS* cardiovascular diseases (diabetes, dyslipidemia, stroke, or heart diseases), Psychiatric diseases: depression or anxiety, *BMI* Body Mass Index

### Responses to acute stroke

Two thousand two hundred respondents (78.3%) were aware of calling EMS (84.3% outpatients vs 76.0% public, *p* = 0.119), and 93.1% were aware of seeking immediate medical assistance by stroke attack (94.3% outpatients vs 92.5% public, *p* = 0.754). Odds of calling EMS immediately by respondents was 3.8 times higher than of calling EMS not immediately (*p* < 0.001) (Table 2). Similarly, for public and outpatients, odds were 3 and 8.1 times higher respectively.

**Table 2:** Awareness of calling EMS and seeking immediate medical assistance among respondents

	Calling EMS	Seeking immediate medical assistance			OR
	n(%)	Yes	No	***p***	
		n(%)	n(%)		
**Total respodents**				< 0.001	3.8
Aware	2200(78.3)	2099(97.0)	101(51.8)		
Unaware	608(21.7)	514(3.0)	94(48.2)		
**Public**				< 0.001	3
Aware	1530(76.0)	1449(77.8)	81(54.0)		
Unaware	483(24.0)	414(22.2)	69(46.0)		
**Outpatients**				< 0.001	8.1
Aware	670(84.3)	650(86.7)	20(44.4)		
Unaware	125(15.7)	100(13.3)	25(55.6)		
	Seeking immediate medical assistance	Calling EMS	No		
		Yes			
**Total respondents**				< 0.001	3.8
Aware	2613(93.1)	2099(95.4)	514(84.5)		
Unaware	195(6.9)	101(4.6)	94(15.5)		
**Public**				< 0.001	3
Aware	1863(92.5)	1449(94.7)	414(85.7)		
Unaware	150(7.5)	81(5.3)	69(14.3)		
**Outpatients**				< 0.001	8.1
Aware	750(94.3)	650(97.0)	100(80.0)		
Unaware	45(5.7)	20(3.0)	25(20.0)		

*EMS* emergency medical services, *OR* odds ratio

For each of the specific stroke symptoms, outpatients and public would contact medical clinic or call EMS without any significance difference between them, even though the majority (about 50%) would rather contact a medical clinic than call the EMS or take other actions (wait and see, no idea, or nothing) (Table 3).

**Table 3:** Acute individual stroke symptom’s responses

	Total	Public	Outpatients	*p*
	*n* = 2808	*n* = 2013	*n* = 795	
	no. aware (% aware)	no. aware (% aware)	no. aware (% aware)	
**Speech impairment**				
Call EMS	1103(39.3)	785(39.0)	318(40.0)	0.788
Contact medical clinic	1436(51.1)	1007(50.0)	429(54.0)	0.357
Other	269(9.6)	221(11.0)	48(6.0)	0.004
**Dizziness/ loss of balance**				
Call EMS	793(28.2)	565(28.1)	228(28.7)	0.846
Contact medical clinic	1431(51.0)	1009(50.1)	422(53.1)	0.487
Other	584(20.8)	439(21.8)	145(18.2)	0.178
**Acute headache**				
Call EMS	873(31.1)	624(31.0)	249(31.3)	0.922
Contact medical clinic	1434(51.1)	1012(50.3)	422(53.1)	0.510
Other	501(17.8)	377(18.7)	124(15.6)	0.202
**Blurred/ double vision**				
Call EMS	881(31.4)	602(29.9)	279(35.1)	0.124
Contact medical clinic	1468(52.3)	1024(50.9)	444(55.8)	0.250
Other	459(16.3)	387(19.2)	72(9.1)	< 0.001
**Numbness/ dead sensation on one side of body**				
Call EMS	1226(43.7)	876(43.5)	350(44.0)	0.897
Contact medical clinic	1467(52.2)	1042(51.8)	425(53.5)	0.693
Other	115(4.1)	95(4.7)	20(2.5)	0.049
**Facial muscles weakness on lower part on one side**				
Call EMS	1184(42.2)	840(41.7)	344(43.3)	0.690
Contact medical clinic	1505(53.6)	1078(53.6)	427(53.7)	0.971
Other	119(4.2)	95(4.7)	24(3.0)	0.143
**Weakness on one body side**				
Call EMS	1179(42.0)	853(42.4)	326(41.0)	0.721
Contact medical clinic	1501(53.5)	1051(52.2)	450(56.6)	0.315
Other	128(4.6)	109(5.4)	19(2.4)	0.009
**Confusion**				
Call EMS	701(25.0)	497(24.7)	204(25.7)	0.744
Contact medical clinic	1557(55.4)	1081(53.7)	476(59.9)	0.167
Other	550(19.6)	435(21.6)	115(14.5)	0.004

### Sources of stroke information

A significantly higher percentage of outpatients than the public had television/ radio (66.9% vs 56.2%), and magazines/ newspapers (58.9% vs 38.2%) as sources of information than the public (*p* < 0.05) (eTable 1). The public were most likely to get stroke information from family/ friends (61.2%) and lowest from others (15.7%). Outpatients were most likely to get information from TV/ radio (66.9%) and lowest from others (18.0%).

### Awareness differences of calling EMS when having acute stroke by respondents’ sociodemographic factors and other characteristics

#### Sociodemographic factors

Outpatients had higher awareness than the public for calling EMS when perceiving stroke (*p* < 0.05) among those aged > 50 years (91.1% vs 64.2%), and not residing/working together (93.9% vs 75.8%) (Table 4). The public residing/working together had higher awareness than outpatients for calling EMS (76.2% vs 24.5%, *p* < 0.001).

**Table 4:** Awareness of calling EMS by sociodemographic and stroke risk factors, stroke responses and sources of information

	Public *n* = 2013	no. aware (% aware)	Outpatients no. of respondents	no. aware (% aware)	^*#*^*p*
**Sociodemographic factors**					
**Gender**					
Female	1097	861(78.5)	462	402(87.0)	0.232
Male	916	669(73.0)	333	268(80.5)	0.348
**Age**					
18-34 yrs	1118	886(79.2)	383	306(79.9)	0.931
35-49 yrs	588	447(76.0)	254	220(86.6)	0.268
> 50 yrs	302	194(64.2)	157	143(91.1)	0.028
**Education**					
None/unspecified/ primary	252	165(65.5)	115	101(87.8)	0.108
Secondary	1113	853(76.6)	405	346(85.4)	0.234
Tertiary	648	512(79.0)	275	223(81.1)	0.819
**Medical insurance**					
No	2013	1530(76.0)	375	287(76.5)	0.939
Yes	0	NA	420	383(91.2)	NA
**Marital status**					
Married/cohab	728	546(75.0)	254	218(85.8)	0.241
Other (single, divorcee, widowed, unspecified)	1285	984(76.6)	541	452(83.5)	0.282
**Residing/working together**					
No	1002	760(75.8)	685	643(93.9)	0.005
Yes	1011	770(76.2)	110	27(24.5)	< 0.00001
**Stroke action**					
**Seeking immediate medical assistance by stroke**					
No	150	81(54.0)	45	20(44.4)	0.571
Yes	1863	1449(77.8)	750	650(86.7)	0.109
**Stroke symptoms’ reaction**					
**Speech Impairment**					
EMS	785	713(90.8)	318	298(93.7)	0.750
Medical clinic	1007	686(68.1)	429	356(83.0)	0.036
Other	221	131(59.3)	48	16(33.3)	0.089
**Dizziness/loss of balance**					
EMS	565	527(93.3)	228	214(93.9)	0.957
Medical clinic	1009	730(72.3)	422	368(87.2)	0.042
Other	439	273(62.2)	145	88(60.7)	0.888
**Acute headache**					
EMS	624	577(92.5)	249	231(92.8)	0.976
Medical clinic	1012	706(69.8)	422	354(83.9)	0.049
Other	377	247(65.5)	124	85(68.5)	0.800
**Blurred/double vision**					
EMS	602	566(94.0)	279	262(93.9)	0.991
Medical clinic	1024	710(69.3)	444	373(84.0)	0.037
Other	387	254(65.6)	72	35(48.6)	0.214
**Numbness/dead sensation on one side of the body**					
EMS	876	797(91.0)	350	326(93.1)	0.801
Medical clinic	1042	687(65.9)	425	335(78.8)	0.062
Other	95	46(48.4)	20	9(45.0)	0.886
**Facial muscles weakness on the lower part on one side**					
EMS	840	772(91.9)	344	325(94.5)	0.769
Medical clinic	1078	718(66.6)	427	336(78.7)	0.079
Other	95	40(42.1)	24	9(37.5)	0.821
**Weakness on one body side**					
EMS	853	764(89.6)	326	294(90.2)	0.944
Medical clinic	1051	693(65.9)	450	361(80.2)	0.036
Other	109	73(67.0)	19	15(78.9)	0.690
**Confusion**					
EMS	497	465(93.6)	204	180(88.2)	0.635
Medical clinic	1081	745(68.9)	476	396(83.2)	0.035
Other	435	320(73.6)	115	94(81.7)	0.531
**Self-reported risk factors**					
**History of hypertension**					
No/unspecified	1872	1424(76.1)	660	551(83.5)	0.195
Yes	141	106(75.2)	135	119(88.1)	0.399
**CVDS**					
No	1896	1439(75.9)	716	600(83.8)	0.154
Yes	117	91(77.8)	79	70(88.6)	0.564
**Smoking**					
No/unspecified	1809	1390(76.8)	619	533(86.1)	0.119
Current	182	123(67.6)	155	117(75.5)	0.545
Former	22	17(77.3)	21	20(95.2)	0.653
**Healthy diet**					
No /unspecified	802	570(71.1)	317	288(90.9)	0.019
Yes	1211	960(79.3)	478	382(79.9)	0.924
**Alcohol consumption status**					
None/unspecified	1583	1222(77.2)	511	439(85.9)	0.180
Current	406	287(70.7)	262	216(82.4)	0.230
Former	24	21(87.5)	22	15(68.2)	0.599
**Family history of stroke/heart diseases**					
None/unspecified	1246	997(80.0)	454	360(79.3)	0.917
Both stroke and heart diseases	227	139(61.2)	162	147(90.7)	0.019
Heart diseases	227	158(69.6)	120	108(90.0)	0.152
Stroke	313	236(75.4)	59	55(93.2)	0.333
**History of HIV/AIDS**					
No/unspecified	1724	1267(73.5)	515	409(79.4)	0.340
Yes	289	263(91.0)	280	261(93.2)	0.846
**History of psychiatric diseases**					
No	2013	1530(76.0)	706	658(93.2)	0.002
Yes	0	NA	89	12(13.5)	NA
**Calculated risk factors**					
**Physical activity status (MET min/week)**					
None/unspecified	1585	1185(74.8)	584	511(87.5)	0.039
Light	66	47(71.2)	46	26(56.5)	0.498
Moderate	290	238(82.1)	146	122(83.6)	0.909
High	72	60(83.3)	19	11(57.9)	0.405
**BMI status**					
Underweight	85	66(77.6)	20	13(65.0)	0.670
Normal, unknown	904	673(74.4)	274	207(75.5)	0.896
Overweight	458	359(78.4)	211	189(89.6)	0.299
Obesity	566	432(76.3)	290	261(90.0)	0.142
**Source of stroke information**					
Family or friends	1231	951(77.3)	482	414(85.9)	0.208
Tv or radio	1132	845(74.6)	532	479(90.0)	0.022
Newspaper or magazine	769	657(85.4)	468	434(92.7)	0.351
Social Media (Internet, Facebook, WhatsApp)	527	436(82.7)	202	187(92.6)	0.369
Doctor or nurse	754	599(79.4)	316	281(88.9)	0.275
Others (school, patients, experience)	316	249(78.8)	143	112(78.3)	0.970

*NA* not applicable

^#^between outpatients and public

#### Stroke action

Awareness rates of calling EMS by stroke among respondents who would call EMS by any specific stroke symptom was high (at least 88%) for all symptoms. Among those who would call medical clinic when experiencing stroke symptoms, outpatients had higher awareness than the public (*p* < 0.05) for speech impairment (83.0% vs 68.1), dizziness/ loss of balance (87.2% vs 72.3%), acute headache (83.9% vs 69.8%), blurred/ double vision (84.0% vs 69.3%), weakness on one side of the body (80.2% vs 65.9%), and confusion (83.2% vs 68.9%).

#### Respondents’ stroke risk factors

For self-reported risk factors, outpatients with the following characteristics had higher awareness than the public for calling EMS by stroke (*p* < 0.05): unhealthy diet (90.9% vs 71.1%), physical inactivity (87.8% vs 75.0%), family history of both stroke and heart diseases (90.7% vs 61.2%), and no history of psychiatric diseases (93.2% vs 76.0%).

For calculated risk factors, physical inactive outpatients had higher awareness than the public for calling EMS (87.5% vs 74.8%, *p* = 0.039).

#### Source of information for stroke

For those with TV/ radio as source of information, outpatients had higher awareness than the public for calling EMS (90.0% vs 74.6%, *p* = 0.022). Awareness was highest for those with newspaper/ magazine as source of information (85.4% public vs 92.7% outpatients) while lowest for TV/ radio (74.6%) for public, and others (78.3%) for outpatients.

### Awareness differences of seeking immediate medical assistance when having acute stroke by respondents’ sociodemographic factors and other characteristics

There were no significant awareness differences for seeking medical assistance between outpatients and public based on respondents’ sociodemographic or stroke risk factors, responses to acute stroke symptoms, or sources of stroke (Table 5).

**Table 5:** Awareness of seeking immediate medical assistance by sociodemographic and stroke risk factors, stroke responses and source of information

	Public *n* = 2013	no. aware (% aware)	Outpatients *n* = 795	no. aware (% aware)	^*#*^*p*
**Sociodemographic factors**					
**Gender**					
Female	1097	1023(93.3)	462	437(94.6)	0.861
Male	916	840(91.7)	333	313(94.0)	0.793
**Age**					
18-34 yrs	1118	1021(91.3)	383	357(93.2)	0.814
35-49 yrs	588	551(93.7)	254	243(95.7)	0.849
> 50 yrs	302	287(95.0)	157	149(94.9)	0.992
**Education**					
None/unspecified/primary	252	234(92.9)		107(93.0)	0.990
Secondary	1113	1011(90.8)	405	377(93.1)	0.775
Tertiary	648	618(95.4)	275	266(96.7)	0.892
**Medical insurance**					
No	2013	1863(92.5)	375	345(92.0)	0.943
Yes	0	0(0.0)	420	405(96.4)	NA
**Marital status**					
Married/cohab	728	671(92.2)	254	239(94.1)	0.847
Other (single, divorcee, widowed, unspecified)	1285	1192(92.8)	541	511(94.5)	0.809
**Residing/working status**					
Not from same company/ compound/ family	1002	946(94.4)	685	659(96.2)	0.793
Same	1011	917(90.7)	110	91(82.7)	0.546
**Stroke action**					
**Awareness of calling EMS**					
No	483	414(85.7)	125	100(80.0)	0.658
Yes	1530	1449(94.7)	670	650(97.0)	0.719
**Stroke symptoms’ reaction**					
**Speech Impairment**					
EMS	785	728(92.7)	318	302(95.0)	0.807
Medical clinic	1007	924(96.7)	429	414(96.5)	0.978
Other	221	161(72.9)	48	34(70.8)	0.916
**Dizziness/Loss of Balance**					
EMS	565	546(96.6)	228	224(98.2)	0.883
Medical clinic	1009	955(94.6)	422	404(95.7)	0.892
Other	439	362(82.5)	145	122(84.1)	0.892
**Acute Headache**					
EMS	624	586(93.9)	249	242(97.2)	0.752
Medical clinic	1012	957(94.6)	422	408(96.7)	0.792
Other	377	320(84.9)	124	100(80.6)	0.750
**Blurred/Double Vision**					
EMS	602	575(95.5)	279	267(95.7)	0.985
Medical clinic	1024	982(95.9)	444	426(95.9)	0.995
Other	387	306(79.1)	72	57(79.2)	0.995
**Numbness/Dead Sensation on one side of the body**					
EMS	876	822(93.8)	350	328(93.7)	0.989
Medical clinic	1042	968(92.9)	425	406(95.5)	0.695
Other	95	73(76.8)	20	16(80.0)	0.918
**Facial Muscles weakness on the lower part on one side**					
EMS	840	783(93.2)	344	322(93.6)	0.964
Medical clinic	1078	1009(93.6)	427	409(95.8)	0.781
Other	95	71(74.7)	24	19(79.2)	0.876
**Weakness on one body side**					
EMS	853	789(92.5)	326	307(94.2)	0.851
Medical clinic	1051	987(93.9)	450	429(95.3)	0.854
Other	109	87(79.8)	19	14(73.7)	0.842
**Confusion**					
EMS	497	469(94.4)	204	194(95.1)	0.949
Medical clinic	1081	1022(94.5)	476	456(95.8)	0.869
Other	435	372(85.5)	115	100(87.0)	0.917
**Self-reported risk factors**					
**Hypertension**					
No/unspecified	1872	1733(92.6)	660	623(94.4)	0.769
Yes	141	130(92.2)	135	127(94.1)	0.909
**CVDS**					
No	1896	1755(92.6)	716	675(94.3)	0.775
Yes	117	108(92.3)	79	75(94.9)	0.895
**Smoking**					
No/unspecified	1809	1685(93.1)	619	581(93.9)	0.911
Current	182	160(87.9)	155	150(96.8)	0.551
Former	22	18(81.8)	21	19(90.5)	0.829
**Healthy diet**					
No /unspecified	802	761(94.9)	317	305(96.2)	0.885
Yes	1211	1102(91.0)	478	445(93.1)	0.775
**Alcohol consumption status**					
No/unspecified	1583	1496(94.5)	511	482(94.3)	0.980
Current	406	349(86.0)	262	248(94.7)	0.414
Former	24	18(75.0)	22	20(90.9)	0.675
**Family history of stroke/heart diseases**					
None/unspecified	1246	1171(94.0)	454	424(93.4)	0.937
Both stroke and heart diseases	227	206(90.7)	162	155(95.7)	0.726
Heart diseases	227	212(93.4)	120	116(96.7)	0.833
Stroke	313	274(87.5)	59	55(93.2)	0.766
**History of HIV/AIDS**					
No/unspecified	1724	1616(93.7)	515	487(94.6)	0.904
Yes	289	247(85.5)	280	263(93.9)	0.451
**History of psychiatric diseases**					
No	2013	1863(92.5)	706	674(95.5)	0.626
Yes	0	0(0.0)	89	76(85.4)	NA
**Calculated risk factors**					
**Physical activity status (MET min/week)**					
None/unspecified	1585	1481(93.4)	584	550(94.2)	0.911
Light	66	53(80.3)	46	44(95.7)	0.546
Moderate	290	262(90.3)	149	137(93.8)	0.800
High	72	67(93.1)	19	19(100.0)	0.846
**BMI status**					
Underweight	85	70(82.4)	20	19(95.0)	0.702
Normal, unknown	904	814(90.0)	274	251(91.6)	0.867
Overweight	458	431(94.1)	211	202(95.7)	0.887
Obese	566	548(96.8)	290	278(95.9)	0.924
**Source of information**					
Family or friends	2131	1131(91.9)	482	456(94.6)	0.710
TV or radio	1132	1092(96.5)	532	512(96.2)	0.975
Newspaper or magazine	769	751(97.7)	468	454(97.0)	0.937
Medical doctor or nurse	754	739(98.0)	316	300(94.9)	0.741
Social Media (Internet, Facebook, WhatsApp)	527	519(98.5)	202	199(98.5)	0.998
Others (school, experience, patients)	316	276(87.3)	132	129(90.2)	0.831

*NA* not applicable

^#^between public and outpatients

For both the public and outpatients, awareness of seeking immediate medical assistance was highest for social media (98.5% each) as source of information, and lowest for others (87.3% vs 90.2% respectively).

### Predictors of calling EMS immediately by acute stroke

Outpatients had higher awareness of calling EMS than the public, with mean scores (95% CI) of 0.84(0.82–0.87) vs 0.76(0.74–0.78), *p* < 0.001. Predictors of low awareness of both calling EMS and seeking immediate medical assistance were no medical insurance, residing/working together, history of psychiatric diseases, and normal weight (eTable 2).

Male gender, ≥50 years age, primary education, family history of both stroke and heart diseases, current smoking, no history of HIV/AIDS, and light physical activity were predictors of low awareness of calling EMS, while predictors of low awareness of seeking immediate medical assistance were 18–34 years age, secondary education, family history of stroke, former smokers, former and current drinkers, being on a healthy diet, history of HIV/AIDS, and being underweight.

## Discussion

Our study adds to the meagre literature in Sub-Saharan Africa on awareness of stroke responses and factors influencing them, in addition to comparing outpatients and the public awareness. Awareness of calling EMS or seeking immediate medical assistance was adequate among both respondents. There were some similarities and disparities in predictors of calling EMS and seeking immediate medical assistance by acute stroke.

Awareness of calling EMS by acute stroke was high among both outpatients and the public (84.3% vs 76.0% respectively). Some patients’ studies [12, 13, 18, 20, 21, 24–26, 29, 33, 35, 39, 41, 49, 50] have shown variations and lower rates than ours (15.0–73.0%). This is further supported by one study that reported that time from symptom onset to first call for medical help accounted for 45% of the prehospital delay among stroke patients [15]. Some studies [26, 43, 51–60] conducted among the public also showed some variations in awareness of calling EMS (26.9–89.9%). Some have shown that despite high awareness of stroke symptoms in real life, a significant proportion still fails to call EMS by acute stroke [35, 50, 59, 61]. These discrepancies can be explained by differences in study population (respondents’ age, gender distribution, time and place of study, type of patients, comorbidities) and nature of questions (closed- or open-ended). This could also be due to that stroke as a medical emergency have been emphasized a lot in the past years, therefore, the population has probably better awareness now than in the past.

In contrast, when asked how they would respond to each of the eight stroke symptoms without reference to stroke, awareness rates of calling EMS by any specific stroke symptom was high (at least 88%) for each symptom among both outpatients and public, and without any significant differences between them. This contrasts some studies [7, 43, 60], that had lower awareness rates of calling EMS by blurred/ double vision (23.6–33%), weakness on the body (41.9–59%), speech impairment (41–72.4%), and dead sensation (30.3–51.0%). In addition, other studies showed also lower rates of calling EMS by weakness on one side of the body or speech impairment (44% each), weakness on one lower side of the face (64.3%), dizziness (3.2%) and headache (6.7%) [43, 60, 62]. Discrepancies in these studies can be attributed to differences in study population. Outpatients had higher awareness than the public (*p* < 0.05) for contacting medical clinic by speech impairment (83.0% vs 68.1), dizziness/ loss of balance (87.2% vs 72.3%), acute headache (83.9% vs 69.8%), blurred/ double vision (84.0% vs 69.3%), weakness on one side of the body (80.2% vs 65.9%), and confusion (83.2% vs 68.9%). This could be explained by lack of awareness of EMSs existence since they are mostly available in urban areas, and that outpatients are more frequently in contact with the healthcare system.

The public had as highest source of information family/ friends (61.2%), followed by TV/ radio (56.2%). Outpatients had as highest source of information TV/ radio (66.9%), followed by family/ friends (60.6%). This resonates well with other studies [25, 42, 43, 49, 54, 62, 63] that reported doctors or healthcare professionals as one of the lowest sources of information among patients and public. Highest sources of information at over 40% among outpatients was TV/ radio, family/ friends, and magazines/ newspapers, while for the public it was family/ friends, and TV/ radio. This is supported by other studies [42, 43, 49, 54, 62, 63] that reported mass media, family, and friends as highest sources of stroke information.

Outpatients had higher awareness of calling EMS than the public among those with the following characteristics: age > 50 years, not residing/working together, unhealthy diet, family history of both stroke and heart diseases, no history of psychiatric diseases, calculated physical inactivity, and having TV/radio as source of stroke information. The differences could be explained by patients being more frequently in contact with healthcare professionals, well informed about stroke, also by that most of the public is not aware of EM services exist since they are found mostly in urban areas but not rural areas. However, awareness of calling EMS or seeking immediate medical assistance by source of information was more than 70% for the least source, which shows that all sources of information can be used effectively to relay information about stroke to both outpatients and the public.

Our study showed that predictors of low awareness of calling EMS in general were > 50 years age, primary education, and no medical insurance. This is in line with some studies that demonstrated that lower education [51], older age [51], and no medical insurance [29] were low predictors. It contrasts some studies that showed older age [29, 35, 43] were associated with high awareness of calling EMS, but no association with age [24, 33], education [24, 33, 35], and medical insurance [24, 51]. Our study showed association of low awareness with residing/working together. It contrasts a study that showed those living alone [35] were low predictors, while another one did not show any association [24]. Our study showed association of low awareness with male gender, but no association with marital status, or history of cardiovascular diseases. This resonates with one study that showed association of low awareness with male gender [33] but contrasts some that did not show any association with gender [24, 51]. It partly resonates with some that did not show association with prior stroke [24, 29, 33, 35, 64–66]. It contrasts some studies that showed married marital status, history of cholesterol and history of angina [43] were associated with high awareness.

Awareness of seeking immediate medical assistance was adequate among outpatients and the public, with both achieving mean scores of at least 93.0%. In our study, predictors of low awareness of seeking immediate medical assistance were young age. This is in line with some studies [15, 18, 21], but contrasts some that did not show association with age [19, 30, 33, 67, 68]. Our study showed association of low awareness with secondary education and residing/working together. This contrasts one study that did not show association with education [30], while living alone [33] was associated with low awareness. Our study did not show association of awareness with gender, hypertension, and cardiovascular diseases. It resonates with one study [30] that did not show association with gender. However, it contrasts some studies that showed male gender [18, 33], low risk factor levels [15], and no history of cardiovascular diseases [18] were associated with low awareness. Discrepancies in these studies can be attributed to differences in study population.

### Limitations

Our study is one of the very few studies worldwide if not the first in Sub-Saharan Africa assessing awareness of calling EMS and seeking immediate medical assistance by acute stroke among public and outpatients concurrently. All information from the questionnaires was collected through standardized face-to-face interviews. We compared our results with mostly previous closed-ended studies for the public and patients.

There are some limitations to this study. First, the survey was conducted in only communities and healthcare facilities in greater Gaborone and not all communities/ healthcare facilities were represented, therefore it may not represent all communities in the country. Second, despite all similarities and variations between studies, some studies considered better/ high awareness differently with some either considering awareness based on sums of awareness questions while we resorted to lowest or highest mean score. Third, self-reported factors/characteristics are prone to bias. Lastly, there may be differences in demographic and other factors between responders and non-responders that we are unable to account for. Despite these limitations, a reasonable high response rate of 94% was attained and therefore these results represent current knowledge of the public and outpatients in greater Gaborone.

## Conclusion

Despite adequate awareness of calling EMS or seeking immediate medical services by acute stroke, there are still gaps in awareness among some subgroups. Therefore, results call for policy makers together with other stakeholders for educational campaigns on awareness of calling EMS/ seeking immediate medical assistance by stroke targeting these subgroups using all sources of information available.

## Supplementary Information

**Additional file 1: eFigure 1.** Awareness of calling EMS by acute stroke study.

**Additional file 2.**

**Additional file 3: eTable 1.** Sources of stroke information among respondents.

**Additional file 4: eTable 2.** Mann-Whitney U/ Kruskal-Wallis H - Association of awareness of calling EMS, and seeking immediate medical assistance with sociodemographic factors among respondents.

**Additional file 5.**

## Data Availability

The datasets used and analyzed during the current study are available in the attached file.
