# Supplementary material for: Public and outpatients’ awareness of calling emergency medical services immediately by acute stroke in an upper middle-income country: a cross-sectional questionnaire study in greater Gaborone, Botswana
Source: BMC Neurol. 2022 Sep 14;22:347. doi: 10.1186/s12883-022-02859-z (PMC9472421; doi:10.1186/s12883-022-02859-z)
Supplement: Supplementary file 1 — Additional file 1: eFigure 1. Awareness of calling EMS by acute stroke study. [file 12883_2022_2859_MOESM1_ESM.docx]

| **eFigure 1. Awareness of calling EMS by acute stroke study** |
| --- |

**Q1. Patient sociodemographic factors**

| **Gender** | \| **M** \| **F** \| \| --- \| --- \| | **Medical insurance** | \| **Yes** \| **No** \| \| --- \| --- \| |
| --- | --- | --- | --- | --- | --- | --- | --- |
| **Age (years)** |  | **Residing/working together** | \| **Yes** \| **No** \| \| --- \| --- \| |
| **Education** | \| **Primary** \| **Secondary** \| \| --- \| --- \| \| **Tertiary** \| **None** \| | **Marital status** | \| **Married** \| **Single** \| \| --- \| --- \| \| **Cohabiting** \| **Widowed/divorced** \| |
| **Location** | \| **Scot LH** \| **Ram V** \| \| --- \| --- \| \| **Ram RC** \| **Gabz** \| \| **Mosh C** \| **Mosh V** \| \| **Sbrana** \| **Puth C** \| \| **Nko C** \|  \| |  |  |

**Scot LH=Scottish Livingstone Hospital Ram RC=Ramotswa Railway Station clinic**

**Mosh C=Moshupa Clinic Mosh V =Moshupa village Gabz= Gaborone city**

**Sbrana=Sbrana Psychiatric Referral Hospital Nko C=Nkoyaphiri clinic**

**Puth C=Phuthadikobo clinic Ram V=Ramotswa village**

| **Q2a:** **What would you do when you suspect you are having stroke?**  **Cross only one answer below** | |
| --- | --- |
|  |  |
| 1.Call 911/997/8 OR EMS |  |
| 2.Call family member |  |
| 3.Contact traditional doctor |  |
| 4.Go to the pharmacy |  |
| 5.No idea |  |
| 6.Nothing |  |
| 7.Wait and see |  |

| **Q2b. If you get stroke, how long would you take before seeking medical assistance? Cross only one answer below** | |
| --- | --- |
| **1). Immediately** |  |
| **2). 7 hours** |  |
| **3). 1 day** |  |
| **4). 3 days** |  |
| **5). 1 week** |  |
| **6). No idea** |  |
|  |  |

| **Q2c. What would you do if you get the following? Cross only one answer for each section** | | | | |
| --- | --- | --- | --- | --- |
|  | **Contact medical clinic** | **Nothing** | **Call EMS** | **Wait and see** |
| **1.Speech impairment** |  |  |  |  |
| **2. Dizziness/ loss of balance** |  |  |  |  |
| **3. Acute headache** |  |  |  |  |
| **4. Blurred/ double vision** |  |  |  |  |
| **5. Numbness/ dead sensation on one side of body** |  |  |  |  |
| **6. Facial muscles weakness on lower part on one side** |  |  |  |  |
| **7. Confusion** |  |  |  |  |
| **8. Weakness on one body side** |  |  |  |  |

**Q3a. Self-reporting (or medical reports). Do you have any stroke risk factors?**

|  | **Medical records** | **Self-reported** |  | **Medical records** | **Self-reported** |
| --- | --- | --- | --- | --- | --- |
| **Hypertension** | \| **Yes** \| **No** \| \| --- \| --- \| | \| **Yes** \| **No** \| \| --- \| --- \| | **Sedentary lifestyle** | \| **Yes** \| **No** \| \| --- \| --- \| | \| **Yes** \| **No** \| \| --- \| --- \| |
| **Diabetes** | \| **Yes** \| **No** \| \| --- \| --- \| | \| **Yes** \| **No** \| \| --- \| --- \| | **Smoking** | \| **Yes** \| **No** \| **Ex** \| \| --- \| --- \| --- \| | \| **Yes** \| **No** \| **Ex** \| \| --- \| --- \| --- \| |
| **Dyslipidemia** | \| **Yes** \| **No** \| \| --- \| --- \| | \| **Yes** \| **No** \| \| --- \| --- \| | **Heavy alcohol drinking** | \| **Yes** \| **No** \| **Ex** \| \| --- \| --- \| --- \| | \| **Yes** \| **No** \| **Ex** \| \| --- \| --- \| --- \| |
| **Heart diseases** | \| **Yes** \| **No** \| \| --- \| --- \| |  | **Previous stroke** | \| **Yes** \| **No** \| \| --- \| --- \| | \| **Yes** \| **No** \| \| --- \| --- \| |
| **Family history of stroke** | \| **Yes** \| **No** \| \| --- \| --- \| | \| **Yes** \| **No** \| \| --- \| --- \| | **Family history of both stroke and heart diseases** | \| **Yes** \| **No** \| \| --- \| --- \| | \| **Yes** \| **No** \| \| --- \| --- \| |
| **Family history of heart diseases** | \| **Yes** \| **No** \| \| --- \| --- \| | \| **Yes** \| **No** \| \| --- \| --- \| | **Obesity** | \| **Yes** \| **No** \| \| --- \| --- \| | \| **Yes** \| **No** \| \| --- \| --- \| |
| **HIV/AIDS**  **Other risk factors:**  **1……………**  **2……………**  **3……………** | \| **Yes** \| **No** \| \| --- \| --- \|  \| **Yes** \| **No** \| \| --- \| --- \| \| **Yes** \| **No** \| \| **Yes** \| **No** \| | \| **Yes** \| **No** \| \| --- \| --- \|  \| **Yes** \| **No** \| \| --- \| --- \| \| **Yes** \| **No** \| \| **Yes** \| **No** \| | **Psychiatric disease**  **In case yes, which ones do you have?**  **…………………**  **………………….** | \| **Yes** \| **No** \| \| --- \| --- \|  \| **Yes** \| **No** \| \| --- \| --- \| \| **Yes** \| **No** \| | \| **Yes** \| **No** \| \| --- \| --- \|  \| **Yes** \| **No** \| \| --- \| --- \| \| **Yes** \| **No** \| |

| **What do you think of your weight?** | \| **No idea** \| **Normal** \| **Obese** \| \| --- \| --- \| --- \| | \| **Underweight** \| **Overweight** \| \| --- \| --- \| |
| --- | --- | --- | --- | --- | --- | --- | --- |
| **Do you think you eat healthy?** | \| **Yes** \| **No** \| **No idea** \| \| --- \| --- \| --- \| | **Height 1 (cm):**  **Height 2 (cm):**  **Weight (kg):** |

**Q3b. Physical activities**

| **Do you do any physical activity?** | \| **Yes** \| **No** \| \| --- \| --- \| |
| --- | --- | --- | --- |
| **In case yes, what type of physical activities do you do?** | \| **1.** \| **2.** \| \| --- \| --- \| \| **3.** \| **4.** \| |
| **How many times a day?** | \| **1.** \| **2.** \| \| --- \| --- \| \| **3.** \| **4.** \| |
| **How many minutes in a day?** | \| **1.** \| **2.** \| \| --- \| --- \| \| **3.** \| **4.** \| |
| **How many times in a week?** | \| **1.** \| **2.** \| \| --- \| --- \| \| **3.** \| **4.** \| |
| **How will you grade the intensity of your physical activity?** | \| **1. Inactive** \| **2. Low** \| \| --- \| --- \| \| **3. Moderate** \| **4. High** \| \| **5. No idea** \|  \| |

**Q4. Sources of stroke information**

| How did you get to know about stroke? (*closed-ended question*). Multiple answers  ***MENTION THEM TO THE RESPONDENTS*** | |
| --- | --- |
|  | Cross right answers |
| **TV/ radio** |  |
| **Newspaper/ magazines** |  |
| **Family/ friends** |  |
| **Doctors/ nurses** |  |
| **Social media (internet, Whatsapp, facebook, Instagram, etc)** |  |
| **Others (school, experience, patients)** |  |
