# Supplementary figures and images for: Public and outpatients’ awareness of calling emergency medical services immediately by acute stroke in an upper middle-income country: a cross-sectional questionnaire study in greater Gaborone, Botswana
Source: BMC Neurol. 2022 Sep 14;22:347. doi: 10.1186/s12883-022-02859-z (PMC9472421; doi:10.1186/s12883-022-02859-z)

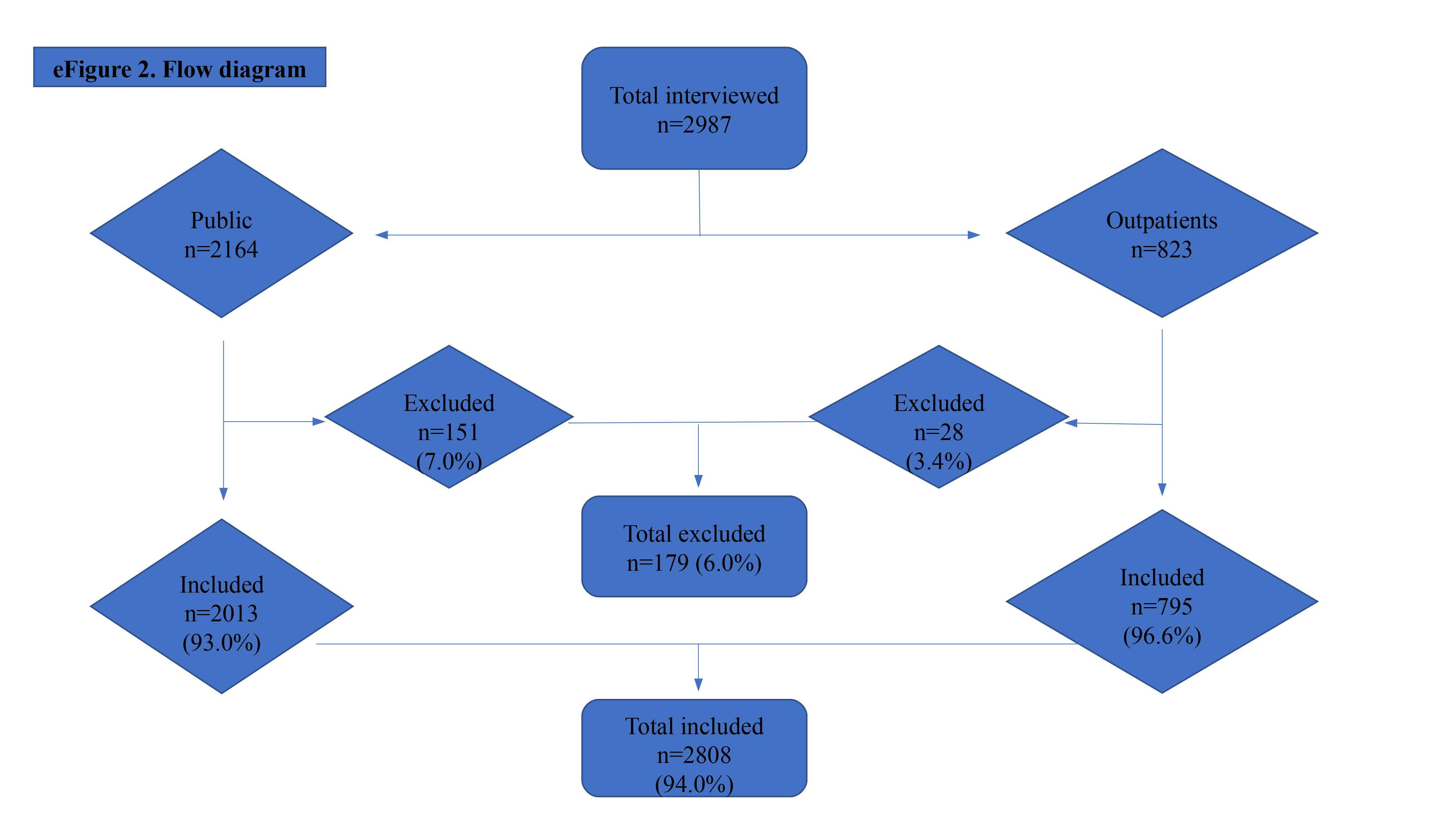

Supplement: Supplementary file 2 — Additional file 2. [file 12883_2022_2859_MOESM2_ESM.jpg]
