# Supplementary material for: Public and outpatients’ awareness of calling emergency medical services immediately by acute stroke in an upper middle-income country: a cross-sectional questionnaire study in greater Gaborone, Botswana
Source: BMC Neurol. 2022 Sep 14;22:347. doi: 10.1186/s12883-022-02859-z (PMC9472421; doi:10.1186/s12883-022-02859-z)
Supplement: Supplementary file 3 — Additional file 3: eTable 1. Sources of stroke information among respondents. [file 12883_2022_2859_MOESM3_ESM.docx]

|  |  |  |  |  |
| --- | --- | --- | --- | --- |
| **eTable 1. Sources of stroke information among respondents** | | | |  |
|  |  |  |  |  |
|  | **Total** | **Public** | **Outpatients** |  |
|  | n=2808 | n=2013 | n=795 |  |
|  | n(%) | n(%) | n(%) | *p* |
| Family or friends | 1713(61.0) | 1231(61.2) | 482(60.6) | 0.910 |
| Tv or radio | 1664(59.3) | 1132(56.2) | 532(66.9) | 0.021 |
| Newspaper or magazine | 1237(44.1) | 769(38.2) | 468(58.9) | <0.001 |
| Doctor or nurse | 1070(38.1) | 754(37.5) | 316(39.7) | 0.533 |
| Social Media (Internet, Facebook, WhatsApp) | 729(26.0) | 527(26.2) | 202(25.4) | 0.798 |
| Others (school, patients, experience) | 459(16.3) | 316(15.7) | 143(18.0) | 0.346 |
|  |  |  |  |  |
|  | |  |  |  |
