# Supplementary material for: Public and outpatients’ awareness of calling emergency medical services immediately by acute stroke in an upper middle-income country: a cross-sectional questionnaire study in greater Gaborone, Botswana
Source: BMC Neurol. 2022 Sep 14;22:347. doi: 10.1186/s12883-022-02859-z (PMC9472421; doi:10.1186/s12883-022-02859-z)
Supplement: Supplementary file 4 — Additional file 4: eTable 2. Mann-Whitney U/ Kruskal-Wallis H - Association of awareness of calling EMS, and seeking immediate medical assistance with sociodemographic factors among respondents. [file 12883_2022_2859_MOESM4_ESM.docx]

|  |  |  |
| --- | --- | --- |
| **eTable 2. Mann-Whitney U/ Kruskal-Wallis H - Association of awareness of calling EMS, and seeking immediate medical assistance with sociodemographic factors among respondents** | | |
|  |  |  |
|  | **Calling EMS** | **Seeking immediate medical assistance** |
|  | Mean (95% CI) | Mean (95% CI) |
| **Sociodemographic factors** |  |  |
| **Gender** |  |  |
| Female | 0.81(0.79-0.83) | 0.94(0.92-0.95) |
| Male | 0.75(0.73-0.77) | 0.92(0.91-0.94) |
| *p* | <0.001 | 0.166 |
| d | 0.072 |  |
|  |  |  |
| **Age** |  |  |
| 1. 18-34yrs | 0.79(0.77-0.81) | 0.92(0.90-0.93) |
| 2. 35-49yrs | 0.79(0.76-0.82) | 0.94(0.93-0.96) |
| 3. >50yrs | 0.73(0.69-0.77) | 0.95(0.93-0.97) |
| *p* | 0.019;  1 vs 3=0.019, 2 vs 3=0.046 | 0.016 |
| ηp2 | 0.003 | 0.003 |
|  |  |  |
| **Education** |  |  |
| 1. None/ unspecified/ primary | 0.72(0.68-0.77) | 0.93(0.90-0.96) |
| 2. Secondary | 0.79(0.77-0.81) | 0.91(0.90-0.93) |
| 3. Tertiary | 0.80(0.77-0.82) | 0.96(0.94-0.97) |
| *p* | 0.013; 1 vs 2=0.020, 1 vs 3=0.015 | <0.001; 2 vs 3<0.001 |
| ηp2 | 0.003 | 0.006 |
|  |  |  |
| **Medical insurance** |  |  |
| No | 0.76(0.74-0.78) | 0.92(0.91-0.94) |
| Yes | 0.91(0.88-0.94) | 0.96(0.95-0.98) |
| *p* | <0.001 | 0.003 |
| d | 0.131 | 0.056 |
|  |  |  |
| **Marital status** |  |  |
| Married/cohab | 0.78(0.75-0.80) | 0.93(0.91-0.94) |
| Others | 0.79(0.77-0.81) | 0.93(0.92-0.94) |
| *p* | 0.606 | 0.554 |
|  |  |  |
| **Residing/working together** |  |  |
| No | 0.83(0.81-0.85) | 0.95(0.94-0.96) |
| Yes | 0.71(0.68-0.74) | 0.90(0.88-0.92) |
| *p* | <0.001 | <0.001 |
| d | 0.003 | 0.101 |
|  |  |  |
| **Respondents self-reported risk factors** | |  |
| **History of hypertension** |  |  |
| No/unspecified | 0.78(0.76-0.80) | 0.93(0.92-0.94) |
| Yes | 0.82(0.77-0.86) | 0.93(0.90-0.96) |
| *p* | 0.178 | 0.967 |
|  |  |  |
| **History of CVDS** |  |  |
| No | 0.78(0.76-0.80) | 0.93(0.92-0.94) |
| Yes | 0.82(0.77-0.88) | 0.93(0.90-0.97) |
| *p* | 0.181 | 0.859 |
|  |  |  |
| **Family history of stroke/heart diseases** | |  |
| 1. No/ unspecified | 0.80(0.78-0.82) | 0.94(0.93-0.95) |
| 2. Both stroke and heart diseases | 0.74(0.69-0.78) | 0.93(0.90-0.95) |
| 3. Heart diseases | 0.77(0.72-0.81) | 0.95(0.92-0.97) |
| 4. Stroke | 0.78(0.74-0.82) | 0.88(0.85-0.92) |
| *p* | 0.044; 1 vs 2=0.039 | 0.002; 1 vs 4=0.001, 3 vs 4=0.008 |
| ηp2 | 0.003 | 0.005 |
|  |  |  |
| **Smoking** |  |  |
| 1. No/ unspecified | 0.79(0.78-0.81) | 0.93(0.92-0.94) |
| 2. Current | 0.71(0.66-0.76) | 0.92(0.89-0.95) |
| 3. Former | 0.86(0.75-0.97) | 0.86(0.75-0.97) |
| *p* | 0.002; 1 vs 2=0.003 | 0.126 |
| ηp2 | 0.005 |  |
|  |  |  |
| **Alcohol consumption status** |  |  |
| 1. No/ unspecified | 0.79(0.78-0.81) | 0.94(0.93-0.95) |
| 2. Current | 0.75(0.72-0.79) | 0.89(0.87-0.92) |
| 3. Former | 0.78(0.66-0.91) | 0.83(0.71-0.94) |
| *p* | 0.089 | <0.001; 1 vs 2<0.001, 1 vs 3=0.005 |
| ηp2 |  | 0.01 |
|  |  |  |
| **Healthy diet** |  |  |
| No /unspecified | 0.77(0.74-0.79) | 0.95(0.94-0.97) |
| Yes | 0.79(0.78-0.81) | 0.92(0.90-0.93) |
| *p* | 0.08 | <0.001 |
| d |  | 0.071 |
|  |  |  |
| **History of HIV/AIDS** |  |  |
| No/unspecified | 0.75(0.73-0.77) | 0.94(0.93-0.95) |
| Yes | 0.92(0.90-0.94) | 0.90(0.87-0.92) |
| *p* | <0.001 | <0.001 |
| d | 0.168 | 0.068 |
|  |  |  |
| **History of psychiatric diseases** | |  |
| No | 0.80(0.79-0.82) | 0.93(0.92-0.94) |
| Yes | 0.13(0.06-0.21) | 0.85(0.78-0.93) |
| *p* | <0.001 | 0.004 |
| d | 0.285 | 0.055 |
|  |  |  |
| **Calculated respondents' risk factors** | |  |
| **Physical activity intensity (MET min/week)** | |  |
| 1. None/ unspecified | 0.78(0.76-0.80) | 0.94(0.93-0.95) |
| 2. Light | 0.65(0.56-0.74) | 0.87(0.80-0.93) |
| 3. Moderate | 0.83(0.79-0.86) | 0.92(0.89-0.94) |
| 4. High | 0.78(0.69-0.87) | 0.95(0.90-0.99) |
| *p* | 0.001; 1 vs 2=0.007, 2 vs 3<0.001 | 0.017; 1 vs 2=0.026 |
| ηp2 | 0.006 | 0.004 |
|  |  |  |
| **BMI status** |  |  |
| 1. Underweight | 0.75(0.67-0.84) | 0.85(0.78-0.92) |
| 2. Normal, unknown | 0.75(0.72-0.77) | 0.90(0.89-0.92) |
| 3. Overweight | 0.82(0.79-0.85) | 0.95(0.93-0.96) |
| 4. Obesity | 0.81(0.78-0.84) | 0.96(0.95-0.98) |
| *p* | <0.001; 2 vs 4=0.004, 2 vs 3=0.002 | <0.001; 1 vs 3=0.001, 1 vs 4<0.001, 2 vs 3=0.004, 2 vs 4<0.001 |
| ηp2 | 0.007 | 0.015 |
|  |  |  |
| EMS: emergency medical services, NA: not applicable, CVDS: cardiovascular diseases (diabetes, stroke, dyslipidemia, or heart diseases), ηp2: partial Eta squared, d: Cohen's d | | |
|  |  |  |
|  |  |  |
